# Five SNPs Within the *FGF5* Gene Significantly Affect Both Wool Traits and Growth Performance in Fine-Wool Sheep (*Ovis aries*)

**DOI:** 10.3389/fgene.2021.732097

**Published:** 2021-09-29

**Authors:** Haiyu Zhao, Ruixue Hu, Fadi Li, Xiangpeng Yue

**Affiliations:** State Key Laboratory of Grassland Agro-Ecosystems, Key Laboratory of Grassland Livestock Industry Innovation, Ministry of Agriculture and Rural Affairs, Engineering Research Center of Grassland Industry, Ministry of Education, College of Pastoral Agriculture Science and Technology, School of Life Sciences, Lanzhou University, Lanzhou, China

**Keywords:** sheep, *FGF5* gene, single nucleotide polymorphism, wool quality traits, growth performance, body weight, association

## Abstract

Fibroblast growth factor 5 (*FGF5*) gene, a member of fibroblast growth factor superfamily, plays significant roles in the regulation of the hair growth cycle during the development of mammalian hair follicles as well as the skeletal muscle development. In this study, DNA sequencing was used to scan the putative SNPs within the full-length of *FGF5* gene, and SNPscan high-throughput technique was applied in the individual genotyping of 604 crossbred sheep. 10 SNPs were identified within *FGF5* gene while five of them located in intron 1 could be genotyped, namely SNP1 (g. 105914953 G > A), SNP2 (g. 105922232 T > C), SNP3 (g. 105922244 A > G), SNP4 (g. 105922334 A > T) and SNP5 (g. 105922340 G > T). All these SNPs were in accord with the Hardy-Weinberg equilibrium (*P* > 0.05), and displayed the moderate polymorphism with PIC values ranging from 0.302 to 0.374. Thereafter, the correlation analysis between each SNP locus and economic traits including wool length, greasy wool weight and growth performance of sheep was systematically implemented. In our results, SNP1, SNP3, SNP4 and SNP5 were significantly associated with wool length, greasy wool weight and growth traits of SG sheep (*P* < 0.05); SNP1, SNP2, SNP3, and SNP4 were significantly correlated with wool length and growth traits of SSG sheep (*P* < 0.05). Meanwhile, our study revealed a strong linkage disequilibrium (LD) relationship among these SNPs (*r*^2^ > 0.33), except for SNP3 and SNP4 sites (*r*^2^ = 0.30). Combination genotype analysis showed that combination genotypes were significantly associated with mean fiber diameter of SG (*P* < 0.05), and body weight trait of SSG (*P* < 0.01). The above findings suggested that these SNP loci might affect economic traits synergistically and could be regarded as potential molecular markers for improving both wool production and growth performance of fine-wool sheep, which lay a molecular foundation for the breeding of fine dual-purpose sheep thereby accelerating the pace of sheep breeding.

## Introduction

Sheep, as an important source of wool, mutton and milk production, is economically influential in the textile and meat industry. Thus, during the last decades, to increase the wool and mutton production, extensive efforts have been made to improve the production potential of sheep products by various research/breeding institutions (Rochus et al., 2020; Erdenee et al., 2021). For instance, Merino sheep crossing with local alpine fine-wool sheep can breed an excellent dual-purpose sheep, with good fine wool length and growth performance. In China, South African mutton merino and Gansu alpine fine wool cross developed a new variety, SG crossbred sheep (South African mutton merino ♂× Gansu alpine fine wool ♀, SG); subsequently, South African mutton merino and SG sheep cross bred another variety, SSG crossbred sheep (South African mutton merino ♂× SG ♀, SSG). SG and SSG sheep varieties have demonstrated good adaptabilities to local climate, improves growth rate and undegraded wool quality. However, both their wool production and growth traits still need to be further improved. Compared with traditional cross-breeding techniques, molecular marker-assisted selection (MAS) breeding has the advantages of low cost, obvious effect and short breeding cycle. Therefore, taking full advantages of the MAS method, the exploration of crucial candidate genes and genetic markers related with wool production as well as growth performance is essential for the improvement of SG and SSG sheep varieties.

Single nucleotide polymorphism (SNP) is defined as a single base-pair substitution in the genomic DNA sequence that occurs with high frequency in the population (Vignal et al., 2002). It is regarded as the most preferable DNA marker in genetic and molecular breeding since its tracking availability, ease of interpretation and suitability for various genotyping techniques (Kruglyak, 1997). Many studies conferred that SNP markers could be used for the breeding of livestock, of which the fundamental goal is to use these mutations as useful markers for the prediction of superior animals concerning the desired production traits (Xiang et al., 2018; Bi et al., 2021; Gautason et al., 2021). Nowadays, the candidate gene association study (CGAS) (Moon, 2019) has been widely used to identify genetic variants of candidate genes and their potential association with certain genotype traits (Pryce et al., 2011; Locke et al., 2015). The success of CGAS largely depends on the choice of candidate genes to be studied, of which the biological function should be relevant to the studied traits (Wilkening et al., 2009; Gebreselassie et al., 2020). For the current sheep breeding, the detailed genetic mutation analyses of crucial genes related to wool production and growth traits are far from enough.

Wool traits and growth performance of sheep are complex physiological and biochemical properties influenced by genetics, the environment and nutrition etc. Many signaling pathways and related factors are involved in the regulation of these economic traits. Among them, fibroblast growth factors (FGFs) are a family of factors that play important roles in promoting and regulating the growth of fibroblasts. Fibroblast growth factor 5 (FGF5) has been verified as a famous dominant inhibitor of hair elongation in many studies. For example, FGF5-null mouse exhibited a long hair phenotype (Nesterova et al., 2010; Zhang et al., 2020); loss-of-function mutations within *FGF5* associated with long-hair phenotypes have been described in many mammalian species, including humans (Higgins et al., 2014), cats (Drögemüller et al., 2007), dogs (Cadieu et al., 2009), rabbits (Xu et al., 2020) and donkeys (Legrand et al., 2014). In addition, the mouse *FGF5* gene was reported to be able to inhibit skeletal muscle development in the limb (Clase et al., 2000). All these findings triggered great interest in the roles of *FGF5* gene in breeding improved wool production and growth of animals. However, although *FGF5* gene has been identified to be polymorphic in many species, there is not any causal mutations that have been identified to be associated with wool traits or the growth performance in sheep.

Therefore, in this study, taking full advantage of our well-established hybrid sheep strain, we investigated the genetic variations of *FGF5* gene in SG and SSG sheep groups. The identified SNPs are significantly correlated with both the wool traits and growth performance of sheep, thereby could be used as candidate molecular markers for the breeding of fine dual types sheep thus accelerating the pace of sheep breeding.

## Materials and Methods

All experiment protocols were reviewed and approved by the Ethics Committee of College of Pastoral Agriculture Science and Technology, Lanzhou University (Ethic approval No: 2010-1 and 2010-2). All efforts were taken to minimize animal suffering.

### Sample Collection and Genomic DNA Extraction

In this study, A total of 401 South African mutton merino (♂) × Gansu alpine fine wool (♀) crossbred sheep (SG sheep) and 203 South African mutton merino (♂) × SG (♀) crossbred sheep (SSG sheep) were selected randomly from sheep-breeding farms in Wuwei City, Gansu Province, China. The sheep were yearlings or adults (2∼2.5 years old), and they were all reared in very similar conditions with appropriate environment and suitable feeding. Blood samples of all individuals were collected. Detailed records of the mean fiber diameter (MFD), greasy wool weight (GWW), wool length in five body parts (shoulder, side, thigh, notum, and abdomen) as well as five indicators of growth performance including body weight, body height, body length, chest girth, and shin circumference were available for all selected individuals (as shown in Table 1 and Supplementary Table 1).

**TABLE 1:** The information of sheep and the economic traits measured.

Population	Age	Sex	Sample size	Measured economic traits
SG	Adult	Female	401	Wool length, mean fiber diameter, the greasy wool weight and growth performance
SSG	Yearling	Male	203	Wool length, mean fiber diameter, and growth performance

*SG, South African mutton merino (♂) and Gansu alpine fine wool (♀) crossbred sheep; SSG, South African mutton merino (♂) and SG (♀) crossbred sheep.*

Genomic DNA was extracted from blood samples using the method previously published (Zhao et al., 2013). Their qualities were then assayed using Nanodrop 2000 Spectrophotometer based on the 1.8 < OD260/280 < 2.0 standard. All qualified genomic DNA samples were diluted to the working concentration of 50 ng/μL and stored at −20°C for further experiments.

### Primer Design and DNA Pool Construction

According to the sheep *FGF5* gene sequence (GenBank accession No. NC_019463.2) in the NCBI database, seven pairs of primers (as shown in Supplementary Table 2) were designed for the amplification of the full-length of *FGF5* gene by using Primer Premier 6.0 software. DNA pooling sequencing was used to screen putative SNPs in each amplified fragment. In order to determine the presence of SNPs within the sheep *FGF5* gene, a total of 30 DNA pools were constructed, and each pool was composed of genomic DNA samples derived from 20 sheep individuals.

### SNP Screening and SNPscan Genotyping

To screen genetic variations cost-effectively, putative SNP identification in sheep *FGF5* gene was achieved by sequencing PCR products of the pooled DNA samples. Briefly, the PCR products were validated by 1.0% agarose gel electrophoresis, then purified and sequenced directly using Sanger sequencing by AoKeDingSheng Biotechnology Company (Beijing, China). To screen the candidate SNPs, sequence alignments were carried out by using DNAstar (DNAstar, United States) and chromas (Technelysium Pty Ltd., Australia) softwares. SNPs were identified by the presence of double peaks at each single locus in the chromatograms. Subsequently, quality control analysis was performed on the candidate SNPs, and the qualified SNPs were genotyped in all 604 sheep samples by using the commercial SNPscan^TM^ high-throughput method (Tianhao Biotech, Shanghai, China).

The PCR amplification was performed in the 25.0 μL reaction condition containing 1.0 μL genomic DNA (50 ng/μL), 0.4 μL of each forward/reverse primer (10 pmol/μL), 12.5 μL of 2 × Taq PCR Super Mix (TranGen Biotech, China) and 10.7 μL of ddH_2_O. The PCR amplification protocol contained a pre-denaturation at 95°C for 5 min and denaturation at 94°C for 30 s, followed by annealing for 30 s at the optimal temperature, 35 cycles of elongation at 72°C for 30 s, and a final extension at 72°C for 5 min with subsequent cooling to 4°C.

### Statistical Analysis

Genotypic and allelic frequencies were estimated based on the obtained genotyping results by using Microsoft Excel software. Genetic parameters including the polymorphism information content (PIC), effective allele number (Ne), homozygosity (Ho) and expected heterozygosity (He) were calculated using the Nei’s method (Nei and Roychoudhury, 1974). The allele frequencies of each SNP were calculated for departure from Hardy-Weinberg equilibrium (HWE) by using the χ^2^-test.

According to the characteristics of the source of sheep sample and the effects of gender, age and variety, a statistical analysis model was established as Y_*ijklm*_ = μ + G_*i*_ + s_*j*_ + p_*k*_ + e_*ijklm*_, in which Y_*ijklm*_ refers to the measured value of the phenotypic trait, μ represents the population mean, G_*i*_ is the genotype effect, s_*j*_ is the gender effect, p_*k*_ is the field effect, and e_*ijklm*_ is the random residual. The effect of birth type had no significant impact on the phenotypic value of wool traits and growth performance, therefore it had been leaving out in our linear model (Bromley et al., 2000). SPSS 22.0 software (IBM) was used to analyze the association between the individual genotypes and the phenotype of the wool traits and growth performance, as well as the combination genotype analysis. The least-squares method was set to fit the linear model (LSE) for comparison. The general linear model (GLM) of ANOVA was used to analyze the relationships between the genotypes and economic traits. According to the correlation coefficients (D′/r^2^), the pattern of pairwise Linkage disequilibrium (LD) between SNP loci was estimated and visualized as previously described (Zou et al., 2021). The case of *D*′ = 1 or *r*^2^ = 1 is known as complete LD. Values of D′ < 1; *r*^2^ > 0.33 indicate strong LD.

## Results

### Identification of Five SNPs Within Sheep *FGF5* Gene

**FIGURE 1:**
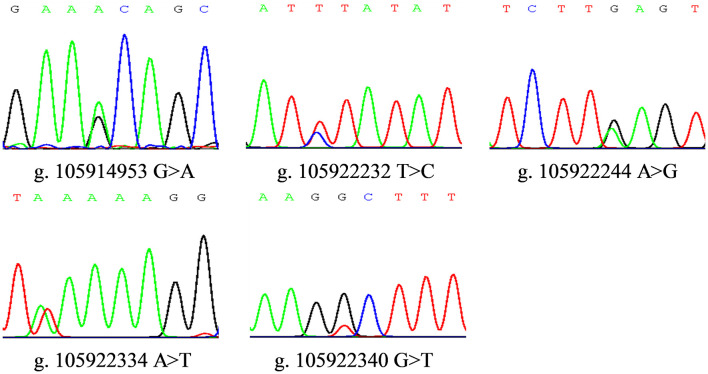
Sequence chromatograms of five SNP loci within sheep *FGF5* gene.

According to our DNA sequencing and sequence alignments results based on 604 sheep samples, 10 putative SNPs were identified within *FGF5* gene while only five of them could be genotyped, namely SNP1 (g. 105914953 G > A), SNP2 (g. 105922232 T > C), SNP3 (g. 105922244 A > G), SNP4 (g. 105922334 A > T) and SNP5 (g. 105922340 G > T). They are all intron mutations located in the first intron of sheep *FGF5* gene. The sequence chromatograms of heterozygous genotypes were illustrated in Figure 1.

### Genotypic and Allelic Frequencies and Population Indexes

Genotypic frequencies, allelic frequencies, as well as the genetic parameters including heterozygosity (He), homozygosity (Ho), effective allele numbers (Ne) and the polymorphism information content (PIC) of five SNP loci within *FGF5* gene in the SG and SSG crossbred sheep group were evaluated (as shown in Table 2 and Supplementary Table 3). The genotypic frequencies of homozygous wild alleles in SNP1, SNP2, SNP3 and SNP5 were all higher than that of mutation alleles, except the SNP4 locus. Based on the χ^2^-test, these five identified SNPs of *FGF5* gene were all in accordance with the Hardy-Weinberg equilibrium (*P* > 0.05). Besides, five SNPs within the *FGF5* gene all displayed moderate polymorphism, with PIC values ranging from 0.302 to 0.374.

**TABLE 2:** The genetic diversity of sheep *FGF5* gene.

SNP Loci	Genotypic frequencies			Allele frequencies		Genetic parameter				
			
	D	H	R	Reference	Mutation	Ho	He	Ne	PIC	HWE (*P*-value)
SNP1	0.570 (342)	0.370 (222)	0.060 (36)	0.755	0.245	0.630	0.370	1.587	0.302	0.997
SNP2	0.341 (204)	0.489 (293)	0.170 (102)	0.585	0.415	0.514	0.486	1.944	0.368	0.854
SNP3	0.547 (328)	0.382 (229)	0.072 (43)	0.738	0.263	0.613	0.387	1.632	0.312	0.727
SNP4	0.220 (130)	0.510 (302)	0.270 (160)	0.475	0.525	0.501	0.499	1.995	0.374	0.577
SNP5	0.484 (275)	0.396 (255)	0.120 (68)	0.682	0.318	0.566	0.434	1.766	0.340	0.448

*Genotype frequency: D, homozygous wild type genotype; H, heterozygous mutant genotype; R, homozygous mutant genotype; Ho, homozygosity; He, heterozygosity; Ne, effective allele numbers; PIC, polymorphism information content; HWE, Hardy–Weinberg equilibrium.*

### Association Analysis of SNPs With Wool Production Traits

The wool traits of the female SG sheep group were recorded from five body parts, namely shoulder, side, thigh, notum, and abdomen. Moreover, the other parameters like mean fiber diameter and greasy wool weight of SG sheep were also recorded in this study. Based on the above information, we explored the correlations between these SNPs of *FGF5* gene and the economic traits. Association analysis showed that the SNP1 (g.105914953 G > A) was significantly correlated with abdomen wool length, and AA carriers showed the best wool length (*P* < 0.05); SNP4 (g.105922334 A > T) was significantly associated with the greasy wool weight, and AA carriers had the best greasy wool weight compared with other carriers (*P* < 0.01); SNP5 (g.105922340 G > T) was also correlated with natural wool length in the abdomen, and GG genotype exhibited significantly improved wool length in comparison with the GT genotype (*P* < 0.05) (Table 3 and Supplementary Table 4).

**TABLE 3:** The correlation analysis of SNPs with wool production related indicators in SG sheep.

Loci	Genotypes	Natural wool length (cm)					MFD (μm)	GWW (kg)
		
		Shoulder	Side	Thigh	Notum	Abdomen		
SNP1	AA	6.17 ± 0.08	6.69 ± 0.10	6.33 ± 0.08	6.37 ± 0.08	**4.67 ± 0.10^a^**	21.63 ± 0.15	3.59 ± 0.05
	GA	6.15 ± 0.09	6.43 ± 0.15	6.21 ± 0.10	6.37 ± 0.12	**4.31 ± 0.11^b^**	21.80 ± 0.20	3.59 ± 0.07
	GG	6.23 ± 0.20	6.25 ± 0.18	6.00 ± 0.12	6.28 ± 0.14	**4.59 ± 0.29^ab^**	21.98 ± 0.49	3.63 ± 0.18
SNP2	CC	6.07 ± 0.08	6.56 ± 0.10	6.23 ± 0.09	6.30 ± 0.08	4.51 ± 0.10	21.59 ± 0.18	3.55 ± 0.06
	CT	6.20 ± 0.09	6.65 ± 0.13	6.30 ± 0.09	6.41 ± 0.11	4.55 ± 0.12	21.81 ± 0.19	3.57 ± 0.06
	TT	6.34 ± 0.18	6.22 ± 0.15	6.20 ± 0.14	6.34 ± 0.16	4.55 ± 0.20	21.76 ± 0.29	3.78 ± 0.11
SNP3	GG	6.13 ± 0.08	6.68 ± 0.10	6.29 ± 0.08	6.36 ± 0.08	4.61 ± 0.09	21.57 ± 0.15	3.63 ± 0.05
	AG	6.22 ± 0.10	6.45 ± 0.14	6.30 ± 0.10	6.38 ± 0.12	4.45 ± 0.13	21.89 ± 0.21	3.54 ± 0.07
	AA	6.21 ± 0.18	6.25 ± 0.15	5.94 ± 0.11	6.24 ± 0.11	4.49 ± 0.29	22.02 ± 0.42	3.64 ± 0.16
SNP4	AA	6.30 ± 0.17	6.35 ± 0.17	6.16 ± 0.14	6.47 ± 0.18	4.60 ± 0.17	21.58 ± 0.28	**3.86 ± 0.10^a^**
	AT	6.18 ± 0.08	6.60 ± 0.12	6.34 ± 0.09	6.39 ± 0.09	4.45 ± 0.10	21.88 ± 0.17	**3.55 ± 0.06^B^**
	TT	6.06 ± 0.09	6.58 ± 0.11	6.18 ± 0.10	6.24 ± 0.09	4.63 ± 0.14	21.52 ± 0.21	**3.50 ± 0.06^B^**
SNP5	GG	6.19 ± 0.09	6.71 ± 0.11	6.28 ± 0.09	6.39 ± 0.09	**4.74 ± 0.12^a^**	21.54 ± 0.16	3.62 ± 0.06
	GT	6.11 ± 0.09	6.42 ± 0.13	6.31 ± 0.10	6.35 ± 0.11	**4.33 ± 0.11^b^**	22.03 ± 0.19	3.53 ± 0.07
	TT	6.30 ± 0.16	6.44 ± 0.16	6.07 ± 0.10	6.31 ± 0.12	**4.53 ± 0.19^ab^**	21.57 ± 0.35	3.73 ± 0.12

*Letters with different genotypes in same trait (a,b/A,B) means significantly at *P* < 0.05/*P* < 0.01, respectively. The bold values indicate significant differences. MFD, mean fiber diameter; GWW, greasy wool weight.*

In addition, the wool length and fiber diameter data of the male SSG sheep group were also available from five body parts, namely shoulder, side, thigh, notum, and abdomen. Our correlation analysis showed that both SNP1 and SNP3 (g.105914953 G > A; g.105922244 A > G) were significantly correlated with thigh wool length (*P* < 0.05); SNP2 (g.105922232 T > C) was significantly associated with the mean fiber diameter (*P* < 0.05); SNP4 (g.105922334 A > T) was correlated with natural wool length in the notum, and AT genotype demonstrated improved wool length compared with other genotypes (*P* < 0.05) (Table 4).

**TABLE 4:** The correlation analysis of SNPs with wool production related indicators in SSG sheep.

Loci	Genotypes	Natural wool length (cm)					MFD (μm)
		
		Shoulder	Side	Thigh	Notum	Abdomen	
SNP1	AA	7.72 ± 0.13	7.95 ± 0.15	**7.58 ± 0.10^b^**	7.99 ± 0.13	5.44 ± 0.11	20.94 ± 0.28
	GA	7.72 ± 0.13	7.93 ± 0.17	**7.48 ± 0.12^b^**	7.87 ± 0.17	5.37 ± 0.13	20.89 ± 0.38
	GG	8.31 ± 0.47	8.64 ± 0.90	**8.53 ± 0.62^a^**	8.83 ± 0.83	5.26 ± 0.55	24.80 ± 0.70
SNP2	CC	7.56 ± 0.18	7.95 ± 0.21	7.54 ± 0.14	7.81 ± 0.18	5.31 ± 0.15	**21.54 ± 0.42^ab^**
	CT	7.79 ± 0.13	8.02 ± 0.15	7.55 ± 0.11	8.04 ± 0.14	5.48 ± 0.11	**20.55 ± 0.29^b^**
	TT	7.91 ± 0.22	7.88 ± 0.28	7.79 ± 0.22	8.05 ± 0.26	5.38 ± 0.22	**21.88 ± 0.57^a^**
SNP3	GG	7.74 ± 0.14	7.93 ± 0.14	**7.60 ± 0.10^ab^**	8.02 ± 0.12	5.47 ± 0.11	21.13 ± 0.28
	AG	7.69 ± 0.13	7.99 ± 0.18	**7.48 ± 0.13^b^**	7.82 ± 0.17	5.36 ± 0.14	20.82 ± 0.40
	AA	8.34 ± 0.44	8.25 ± 0.71	**8.31 ± 0.54^a^**	8.71 ± 0.69	5.03 ± 0.35	20.43 ± 1.67
SNP4	AA	7.93 ± 0.17	7.95 ± 0.20	7.71 ± 0.17	**8.01 ± 0.20^ab^**	5.41 ± 0.17	21.06 ± 0.49
	AT	7.83 ± 0.14	8.12 ± 0.17	7.63 ± 0.11	**8.20 ± 0.15^a^**	5.54 ± 0.12	20.90 ± 0.29
	TT	7.49 ± 0.21	7.84 ± 0.24	7.49 ± 0.15	**7.67 ± 0.19^b^**	5.27 ± 0.17	21.19 ± 0.54
SNP5	GG	7.76 ± 0.15	7.92 ± 0.16	7.66 ± 0.11	8.09 ± 0.14	5.44 ± 0.11	21.25 ± 0.28
	GT	7.70 ± 0.13	8.01 ± 0.17	7.48 ± 0.13	7.84 ± 0.16	5.45 ± 0.13	20.72 ± 0.39
	TT	8.02 ± 0.31	8.03 ± 0.41	7.81 ± 0.27	8.15 ± 0.34	5.01 ± 0.28	20.63 ± 0.82

*Letters with different genotypes in same trait (a,b) means significantly at *P* < 0.05. The bold values indicate significant differences. MFD, mean fiber diameter.*

### Association Analysis of *FGF5* SNPs With Growth Performance

Association analysis demonstrated that the identified SNPs within *FGF5* gene were also significantly correlated with growth traits of female SG sheep, including SNP3 (g.105922244 A > G), SNP4 (g.105922334 A > T) and SNP5 (g.105922340 G > T). SNP3, SNP4, SNP5 had a significant influence on the body height (*P* < 0.05), and AA, AA, and TT carriers had significantly improved body height than that of other carriers, respectively (Table 5). For the male SSG sheep, association analysis demonstrated that SNP1 (g.105914953 G > A), SNP2 (g.105922232 T > C) sites had significant effects on body weight (*P* < 0.05), and GG, CC carriers had improved weight trait than that of other carriers (Table 6).

**TABLE 5:** Correlation analysis of SNPs within the *FGF5* gene with body size in SG sheep.

Loci	Genotypes	Body weight (kg)	Chest girth (cm)	Body height (cm)	Body length (cm)	Shin circumference (cm)
SNP1	AA	38.54 ± 0.42	80.86 ± 0.44	64.33 ± 0.33	67.77 ± 0.45	7.37 ± 0.06
	GA	38.25 ± 0.58	81.59 ± 0.58	64.55 ± 0.36	68.34 ± 0.56	7.37 ± 0.08
	GG	40.51 ± 1.15	82.26 ± 1.14	65.79 ± 0.87	68.63 ± 1.41	7.55 ± 0.16
SNP2	CC	38.44 ± 0.48	80.90 ± 0.51	63.99 ± 0.41	67.37 ± 0.54	7.40 ± 0.08
	CT	38.53 ± 0.50	81.35 ± 0.51	64.68 ± 0.31	68.48 ± 0.50	7.34 ± 0.07
	TT	39.54 ± 0.89	81.97 ± 0.85	65.45 ± 0.72	68.32 ± 0.97	7.52 ± 0.13
SNP3	GG	38.48 ± 0.43	80.85 ± 0.44	**64.16 ± 0.34^b^**	67.68 ± 0.47	7.40 ± 0.07
	AG	38.68 ± 0.57	81.38 ± 0.58	**64.64 ± 0.35^ab^**	68.47 ± 0.55	7.30 ± 0.08
	AA	39.28 ± 1.15	83.14 ± 1.06	**66.10 ± 0.84^a^**	68.33 ± 1.28	7.62 ± 0.16
SNP4	AA	39.59 ± 0.73	81.86 ± 0.83	**65.97 ± 0.65^a^**	69.56 ± 0.92	7.39 ± 0.12
	AT	38.30 ± 0.48	81.23 ± 0.48	**64.20 ± 0.30^b^**	67.81 ± 0.46	7.37 ± 0.07
	TT	38.66 ± 0.55	81.03 ± 0.59	**64.32 ± 0.46^b^**	67.61 ± 0.62	7.40 ± 0.08
SNP5	GG	38.59 ± 0.47	81.05 ± 0.49	**64.38 ± 0.37^ab^**	67.84 ± 0.52	7.41 ± 0.07
	GT	38.29 ± 0.53	81.24 ± 0.54	**64.26 ± 0.32^b^**	67.78 ± 0.50	7.33 ± 0.08
	TT	39.83 ± 0.81	82.06 ± 0.87	**65.81 ± 0.74^a^**	69.58 ± 1.04	7.48 ± 0.12

*Letters with different genotypes in one growth trait (a,b) means significantly at *P* < 0.05. The bold values indicate significant differences.*

**TABLE 6:** Correlation analysis of SNPs within the *FGF5* gene with body size in SSG sheep.

Loci	Genotypes	Body weight (kg)	Chest girth (cm)	Body height (cm)	Body length (cm)	Shin circumference (cm)
SNP1	AA	**47.87 ± 0.74^b^**	92.94 ± 0.67	71.85 ± 0.38	74.60 ± 0.44	9.99 ± 0.09
	GA	**46.87 ± 0.81^b^**	92.77 ± 0.86	71.91 ± 0.39	74.29 ± 0.45	10.08 ± 0.12
	GG	**53.93 ± 3.76^a^**	94.13 ± 3.52	73.75 ± 1.28	76.25 ± 1.70	10.15 ± 0.42
SNP2	CC	**49.78 ± 1.09^a^**	94.51 ± 0.97	72.31 ± 0.53	75.37 ± 0.63	10.19 ± 0.13
	CT	**46.57 ± 0.70^b^**	92.21 ± 0.70	71.72 ± 0.35	74.22 ± 0.43	9.96 ± 0.09
	TT	**47.47 ± 1.37^ab^**	92.44 ± 1.36	72.03 ± 0.67	74.24 ± 0.64	9.99 ± 0.19
SNP3	GG	47.96 ± 0.78	92.94 ± 0.67	72.00 ± 0.37	74.67 ± 0.44	10.01 ± 0.09
	AG	47.31 ± 0.80	93.10 ± 0.88	71.75 ± 0.41	74.33 ± 0.48	10.08 ± 0.12
	AA	47.95 ± 2.84	91.11 ± 2.92	73.00 ± 0.85	74.89 ± 0.99	9.89 ± 0.35
SNP4	AA	47.59 ± 1.09	93.04 ± 0.79	72.15 ± 0.46	74.29 ± 0.55	9.98 ± 0.14
	AT	47.38 ± 0.81	93.04 ± 0.79	72.01 ± 0.39	74.52 ± 0.47	10.05 ± 0.10
	TT	48.28 ± 1.20	93.36 ± 1.14	71.89 ± 0.62	75.00 ± 0.70	10.07 ± 0.14
SNP5	GG	47.85 ± 0.83	92.89 ± 0.76	71.98 ± 0.44	74.85 ± 0.51	10.00 ± 0.09
	GT	47.27 ± 0.82	92.75 ± 0.81	71.98 ± 0.37	74.47 ± 0.42	10.11 ± 0.12
	TT	49.58 ± 1.68	94.12 ± 1.80	71.82 ± 0.67	73.59 ± 0.87	9.85 ± 0.17

*Letters with different genotypes in one growth trait (a,b) means significantly at *P* < 0.05. The bold values indicate significant differences.*

### Linkage Disequilibrium Analysis of SNP Loci Within Sheep *FGF5* Gene

**FIGURE 2:**
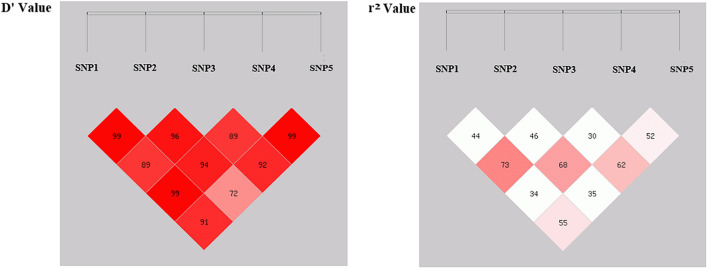
Linkage disequilibrium (LD) analysis of five SNP loci within sheep *FGF5* gene.

In this study, since all these five SNPs were significantly associated with various sheep economic traits, are there any correlation relationships among these SNP loci? To further understand this question, linkage disequilibrium (LD) analysis between SNP1, SNP2, SNP3, SNP4 and SNP5 were analyzed (Figure 2 and Supplementary Table 5). SNP1 had a strong LD relationship with SNP2, SNP3, SNP4 and SNP5 (*r*^2^ > 0.33); SNP2 had a strong LD relationship with SNP1, SNP3, SNP4 and SNP5 (*r*^2^ > 0.33); SNP3 locus had a strong LD relationship with SNP1, SNP2, SNP5 loci (*r*^2^ > 0.33); SNP4 site had a strong LD relationship with SNP1, SNP2 and SNP5 sites (*r*^2^ > 0.33); and SNP5 was strongly linked with SNP1, SNP2, SNP3, and SNP4 sites (*r*^2^ > 0.33). These close links among five loci suggested that there might be a certain synergistic effect on regulating the above economic traits of sheep.

### Combination Genotype Analysis Between Five SNP Loci and Sheep Economic Traits

**FIGURE 3:**
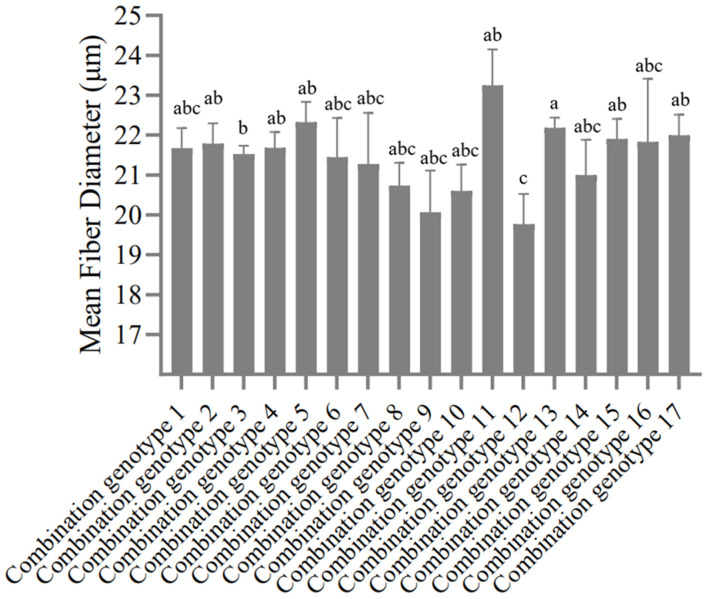
Association analysis between 17 combination genotypes of five SNP loci and Mean Fiber Diameter in SG sheep. Different letters (a,b,c) show significant difference (*P* < 0.05).

**FIGURE 4:**
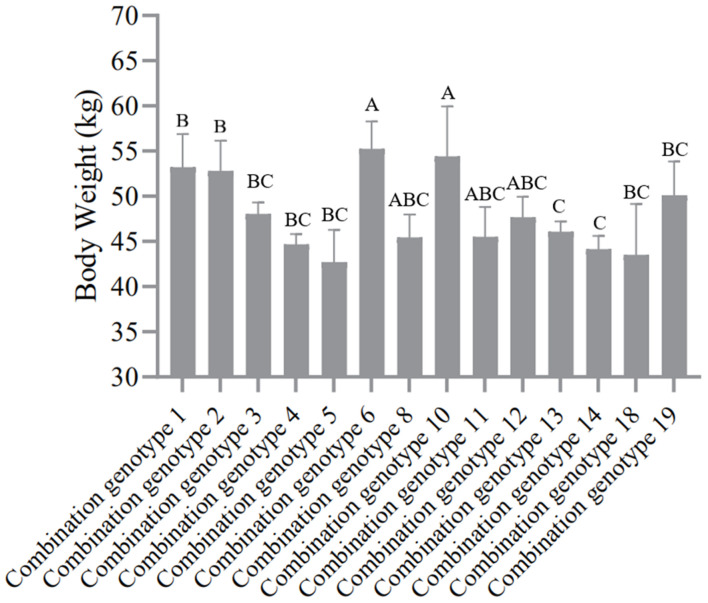
Association analysis between 14 combination genotypes of five SNP loci and Body Weight in SSG sheep. Different letters (A,B,C) show significant difference (*P* < 0.01).

According to the genotype distribution of five SNP sites in SG and SSG sheep population. In the order of SNP1-SNP2-SNP3-SNP4-SNP5, there were 19 combination genotypes. The detailed information of these 19 combination genotypes were shown in Supplementary Table 6. Other combination genotypes which were not present or had only one or two occurrences, were not analyzed. For the SG sheep, the combination genotypes were significantly associated with Mean Fiber Diameter (*P* < 0.05) (Figure 3 and Supplementary Table 7), whereas for the SSG sheep, combination genotypes were significantly corrected with Body Weight trait (*P* < 0.01) (Figure 4 and Supplementary Table 8). Results with no significant correlation between combination genotypes and other phenotypic traits are not shown in this section.

## Discussion

Wool production traits and the growth performance of sheep greatly affect the development of the whole sheep breeding industry. In recent years, to meet the growing needs of the Chinese sheep market, the sheep industry has gradually switched its focus from wool to wool-mutton production. Therefore, it is particularly important to take into account the breeding of improved wool production as well as the growth performance in sheep. Single nucleotide polymorphisms (SNPs) are regarded as the most preferable DNA marker in genetic and molecular breeding since its tracking availability, ease for interpretation, and suitability for genotyping techniques (Kruglyak, 1997; Wang et al., 2019), therefore it has been widely used in the modern genetic breeding of livestock.

In this study, ten SNP mutations within *FGF5* gene were identified in the hybrid SG and SSG sheep while only five of them were focused for genotyping and subsequent association analysis. This is because: (1) technically, only these five SNPs could be genotyped by the probe-based SNPscan method; (2) these five intronic SNPs are adjacent therefore are more likely to exhibit linkage and combined genotype effects; (3) according to previous studies, intronic SNPs as molecular markers also play important roles in the animal genetics and breeding. These intronic SNPs were all consistent with the Hardy-Weinberg equilibrium (*P* > 0.05), and all displayed moderate polymorphism, with a high adaptability under the circumstances of environmental changes. In addition, their associations with wool production and growth traits of sheep were investigated, and our results revealed that these five SNPs within *FGF5* gene were all significantly correlated with sheep economic traits.

For the wool traits of sheep, SNP1, SNP4 and SNP5 were significantly associated with the wool length and greasy wool weight of SG sheep (*P* < 0.05), and SNP1, SNP2, SNP3 and SNP4 were closely correlated with wool length of SSG sheep (*P* < 0.05). These results suggested that all these five SNP mutations might play important roles in the wool production of sheep, and the observed differences of these SNP loci in SG and SSG sheep might be caused by the breed specificity. According to previous studies, the fibroblast growth factor 5, as a member of the fibroblast growth factor superfamily (FGFs), plays an important role in the regulation of the hair growth cycle during the development of mammalian hair follicles (Schneider et al., 2009; Hu et al., 2021). *FGF5* gene could inhibit murine normal hair growth, and its loss of function mutation caused abnormal hair growth by extending the hair growth cycle (Sundberg et al., 1997; Kim et al., 2020). Similar studies also have been conducted in many other species, for example, Hattori et al. (1996) found that hair growth in rat was associated with *FGF5* gene mutation. In humans, previous studies have identified *FGF5* as a crucial gene regulating hair growth and hair loss (Kim et al., 2020). In goats and sheep, CRISPR Cas9 technology has been used to produce functional deletion mutations in *FGF5* gene, and the hair length of mutant individuals was significantly longer than that of the wild type (Hu et al., 2017; Li et al., 2017; Su et al., 2020). All these results have indicated that the presence of *FGF5* gene may affect the hair growth of animals, and the growth of hair could be promoted by altering the expression of *FGF5* gene. In this study, five SNPs within *FGF5* gene identified in Chinese crossbred sheep might significantly influence wool length, which strongly suggests that these genetic variations could be regarded as effective molecular markers for wool sheep breeding.

For the growth performance, SNP3, SNP4 and SNP5 were found to be significantly associate with the body height of SG sheep (*P* < 0.05), and SNP1, SNP2 were significantly correlated with the bodyweight of SSG sheep (*P* < 0.05). These results suggested that these SNPs might influence the growth and development of sheep. Zhang et al. (2015) have cloned the coding sequence of *FGF5*, and qPCR analyses have shown that *FGF5* was widely expressed in various tissues including the skin, muscle, spleen, liver, heart and brain of sheep. In addition, *FGF5* gene has been documented to be able to inhibit mouse skeletal muscle development in the limb (Clase et al., 2000). The ectopic expression of *FGF5* significantly stimulated the tenascin expression, the proliferation and expansion of connective tissue fibroblasts throughout the developing limb, as well as promoted the formation of connective tissue. The above studies strongly support our findings that *FGF5* gene could act as a candidate gene of sheep growth and development. However, further studies are still needed to explore the functional mechanisms of how these SNPs within sheep *FGF5* gene affect the body height and weight of hybrid sheep in our study. Intriguingly, our results pointed out that, in SG sheep, individuals with heterozygous genotypes performed inferiority phenotypes both in the wool length and body weight, leading to a hint that a positive correlation might exist in wool development and body weight. In the Merino sheep, increased live weight was found to result in a moderate correlated increase in wool development (Mortimer et al., 2017). Moreover, weight traits have also been identified to have a moderate positive direct genetic correlation with fleece length in Columbia, Polypay, Rambouillet, and Targhee sheep (Bromley et al., 2000). However, the same effect of heterozygous was not applied to SSG sheep, due to the breed specificity, as well as the limitation of sample size.

Previous studies have well-documented that different gene mutations can be linked with each other thereby affecting the economic traits of animals, such as litter size and growth performance of goats (Wang et al., 2019, 2020). Since all these five SNP loci are significantly associated with the economic traits of sheep, is there a certain synergistic effect among them? Based on this assumption, this study further analyzed the linkage disequilibrium (LD) relationship among these loci and the results revealed that there might be a strong linkage disequilibrium relationship among the five SNPs (*r*^2^ > 0.33), and the r^2^ value between SNP3 and SNP4 was close to the threshold of strong linkage disequilibrium state (*r*^2^ = 0.30), suggesting that all these five SNPs might play a synergistic role in the selection and breeding of sheep with both fine wool traits and improved growth performance. In addition, we performed the combination genotype analysis between different combination genotypes and economic traits of SG and SSG sheep. Interestingly, our results showed that combination genotypes were significantly associated with Mean Fiber Diameter of SG (*P* < 0.05), and Body Weight of SSG (*P* < 0.01), which illustrated that the combined selection of these five SNPs might be meaningful and beneficial to the improvement of economic traits of sheep. Given that there hasn’t been any report on the associations between genetic variations of *FGF5* gene and wool and growth traits in sheep, our study here suggested that functional mutations of *FGF5* might play important roles in both sheep growth and wool production, and these five SNP loci within *FGF5* gene could be used as candidate molecular markers, which is of great significance for the improvement of crossbred sheep breeding.

Limitations of the study: In the present research, we focused on the correlation analysis between *FGF5* SNPs or combination genotypes with various sheep economic traits. We speculate that these linked SNPs may affect the expression of *FGF5* gene thereby modulating wool traits and growth performance of sheep. However, these findings are based mostly on association. Thus, future research on the genotype dependent gene and protein expression levels, especially in hair follicle, muscle and skeleton will provide important insight into the underlying mechanisms.

## Conclusion

In this study, five SNPs loci within the sheep *FGF5* gene were identified in two hybrid sheep strains. Among them, SNP1, SNP3, SNP4 and SNP5 were significantly associated with wool length, greasy wool weight and growth performance of SG sheep, and SNP1, SNP2, SNP3, and SNP4 were significantly correlated with wool length and growth traits of SSG sheep. Meanwhile, there were strong linkage disequilibrium relationships among these SNPs. In addition, combination genotypes were significantly associated with mean fiber diameter of SG, and body weight trait of SSG. These above findings suggested that these newly discovered linked SNPs might be utilized as potential molecular markers in the breeding of dual types of sheep with improved wool traits and growth performance.

## Data Availability Statement

The original contributions presented in the study are included in the article/Supplementary Material, further inquiries can be directed to the corresponding author/s.

## Ethics Statement

The animal study was reviewed and approved by the Ethics Committee of College of Pastoral Agriculture Science and Technology, Lanzhou University (Ethic approval No: 2010-1 and 2010-2).

## Author Contributions

RH performed the experiments. RH and HZ analyzed the data. HZ prepared the manuscript. FL and XY designed the experiments and discussed the results. HZ and XY revised the manuscript. All authors contributed and approved the current submission.

## Conflict of Interest

The authors declare that the research was conducted in the absence of any commercial or financial relationships that could be construed as a potential conflict of interest.

## Publisher’s Note

All claims expressed in this article are solely those of the authors and do not necessarily represent those of their affiliated organizations, or those of the publisher, the editors and the reviewers. Any product that may be evaluated in this article, or claim that may be made by its manufacturer, is not guaranteed or endorsed by the publisher.
