# Supplementary material for: Five SNPs Within the FGF5 Gene Significantly Affect Both Wool Traits and Growth Performance in Fine-Wool Sheep (Ovis aries)
Source: Front Genet. 2021 Sep 29;12:732097. doi: 10.3389/fgene.2021.732097 (PMC8511484; doi:10.3389/fgene.2021.732097)
Supplement: Supplementary file 2 [file Table_2.DOCX]

**Table S6.** The combined genotype information of five SNP loci

| **No.** | **Combination genotypes** | **SNP1** | **SNP2** | **SNP3** | **SNP4** | **SNP5** |
| --- | --- | --- | --- | --- | --- | --- |
| 1 | Combination genotype 1 | AA | CC | GG | AT | GG |
| 2 | Combination genotype 2 | AA | CC | GG | AT | GT |
| 3 | Combination genotype 3 | AA | CC | GG | TT | GG |
| 4 | Combination genotype 4 | AA | CT | GG | AT | GG |
| 5 | Combination genotype 5 | AA | CT | GG | AT | GT |
| 6 | Combination genotype 6 | AA | CT | AG | AT | GT |
| 7 | Combination genotype 7 | AA | CT | AG | TT | GG |
| 8 | Combination genotype 8 | AA | TT | GG | AA | GG |
| 9 | Combination genotype 9 | AA | TT | GG | AA | GT |
| 10 | Combination genotype 10 | GA | CT | GG | AT | GG |
| 11 | Combination genotype 11 | GA | CT | AG | AA | GT |
| 12 | Combination genotype 12 | GA | CT | AG | AA | TT |
| 13 | Combination genotype 13 | GA | CT | AG | AT | GT |
| 14 | Combination genotype 14 | GA | TT | AG | AA | GT |
| 15 | Combination genotype 15 | GA | TT | AG | AA | TT |
| 16 | Combination genotype 16 | GA | TT | AA | AT | GT |
| 17 | Combination genotype 17 | GG | TT | AG | AA | TT |
| 18 | Combination genotype 18 | AA | CT | GG | AA | GT |
| 19 | Combination genotype 19 | GG | TT | AA | AA | TT |
