# Supplementary material for: Five SNPs Within the FGF5 Gene Significantly Affect Both Wool Traits and Growth Performance in Fine-Wool Sheep (Ovis aries)
Source: Front Genet. 2021 Sep 29;12:732097. doi: 10.3389/fgene.2021.732097 (PMC8511484; doi:10.3389/fgene.2021.732097)
Supplement: Supplementary file 6 [file Table_6.DOCX]

**Table S2.** PCR primers used for *FGF5* gene amplification

| **Primer sequence (5′-3′)** | **Gene region** | **Size (bp)** | **Tm/℃** |
| --- | --- | --- | --- |
| F: CAGCAGTAGCACCGTGTCTTC | Exon 1 and part of Intron 1 | 377 | 54 |
| R: ACGCACCTCCAACCCAAC |  |  |  |
| F: TGGTGCTTTCAGTGTCTT | Exon 2 and part of Intron 1 | 636 | 57.6 |
| R: AACTCCTCGTATTCCTAC |  |  |  |
| F: TTATCAGCACCAGGAAGC | Exon 3 and part of Intron 2 | 720 | 57.7 |
| R: ATCTTGGCAGAAAGTGGG |  |  |  |
| F: ACACGGTGAAATACAGAC | Exon 3 | 616 | 50 |
| R: AGTGCAATGAATGAGAAC |  |  |  |
| F: CTTGAGGCATAGGTGGTA | Promoter region | 718 | 52 |
| R: TTGGTAATCTTCCGTGGC |  |  |  |
| F: AGCCACGGAAGATTACCA | Promoter region | 756 | 62 |
| R: AAGGATTCGCAGCCCAAC |  |  |  |
| F: GAGACTGGATACTGGGTAGGCG | Promoter region | 579 | 62.8 |
| R: TGCATCTTGCAGGGTTGG |  |  |  |
